# The Effects of Irreversible Electroporation (IRE) on Nerves

**DOI:** 10.1371/journal.pone.0018831

**Published:** 2011-04-14

**Authors:** Wei Li, Qingyu Fan, Zhenwei Ji, Xiuchun Qiu, Zhao Li

**Affiliations:** Orthopedics Oncology Institute of Chinese PLA, Tangdu Hospital, The Fourth Military Medical University, Xi’an, Shanxi, China; University of Chicago, United States of America

## Abstract

**Background:**

If a critical nerve is circumferentially involved with tumor, radical surgery intended to cure the cancer must sacrifice the nerve. Loss of critical nerves may lead to serious consequences. In spite of the impressive technical advancements in nerve reconstruction, complete recovery and normalization of nerve function is difficult to achieve. Though irreversible electroporation (IRE) might be a promising choice to treat tumors near or involved critical nerve, the pathophysiology of the nerve after IRE treatment has not be clearly defined.

**Methods:**

We applied IRE directly to a rat sciatic nerve to study the long term effects of IRE on the nerve. A sequence of 10 square pulses of 3800 V/cm, each 100 µs long was applied directly to rat sciatic nerves. In each animal of group I (IRE) the procedure was applied to produce a treated length of about 10 mm. In each animal of group II (Control) the electrodes were only applied directly on the sciatic nerve for the same time. Electrophysiological, histological, and functional studies were performed on immediately after and 3 days, 1 week, 3, 5, 7 and 10 weeks following surgery.

**Findings:**

Electrophysiological, histological, and functional results show the nerve treated with IRE can attain full recovery after 7 weeks.

**Conclusion:**

This finding is indicative of the preservation of nerve involving malignant tumors with respect to the application of IRE pulses to ablate tumors completely. In summary, IRE may be a promising treatment tool for any tumor involving nerves.

## Introduction

If a critical nerve is circumferentially involved with tumor, radical surgery intended to cure cancer must sacrifice the nerves [1]. Loss of critical nerves may lead to serious consequences. For example, patients with facial paralysis suffer functional, cosmetic, and psychological problems which impair the ability to communicate [2]. In order to better preserve quality of life after surgery, nerve grafting will be required to bridge the gap between two ends of a nerve after cancer resection [3]. Unfortunately, in spite of the impressive technical advancements in nerve reconstruction, complete recovery and normalization of nerve function is difficult to achieve [4], [5].

Numerous techniques, including chemical (ethanol or acetic acid) ablation, and thermal therapies, such as radiofrequency, laser, microwave, ultrasound, and cryoablation, have been developed for tumor ablation [6], [7]. Irreversible electroporation (IRE) has begun receiving more and more attention as an new local tissue ablation technique in tumor treatment. IRE is a modality in which microsecond electrical pulses are applied across the cell to generate a destabilizing electric potential causing formation of permanent nanoscale defects in the cell membrane. The permanent permeability of the cell membrane leads to changes in cell homeostasis and cell death [8], [9]. IRE is counted as a potential approach without the known complications of other conventional thermal ablative methods which include, non-uniform destruction, protection of tumor by the heat sink effect next to large vessels, destruction of tissue collagen with associated destruction of normal structures [10]. IRE has been tested in humans for lung, prostate, kidney, and liver cancers [11], [12], [13]. Human treatment has revealed that IRE is a feasible and safe technique which could offer some potential advantages over current thermal ablative techniques. [13]. G. Onik et al applied in vivo IRE to canine prostates by means of percutaneous needle electrodes. They found that the nerves were apparently not affected despite the fact that complete areas were covered by IRE electric field [14]. IRE ablation can achieve tumor cell death and preservation of the nerve. This suggests that IRE might be a promising choice to treat tumors near or involved critical nerve. Abramov et al. found that 3 hours after non-thermal 150 V/cm shocks (4 ms long) on rat sciatic nerve the loss of normal action potential amplitude and conduction velocity hadn’t recovered [15]. For the typical IRE treatment involves a higher electric field, it is possible that IRE has a more significant impact on the nerve. The pathophysiology of the nerve after IRE treatment has not been clearly defined. We applied IRE directly to rat sciatic nerves in order to study the long term effects of IRE on the nerve.

## Results

There were no differences between two groups immediately after injury, and nerve continuity was preserved. Complete flaccid paralysis of the operative foot was observed following IRE injury. All animals that were performed surgeries survived before or after the procedure, with no wound infection. After 7 weeks the operative foot regained normal function.

### Behavioral analysis

**Figure 1:**
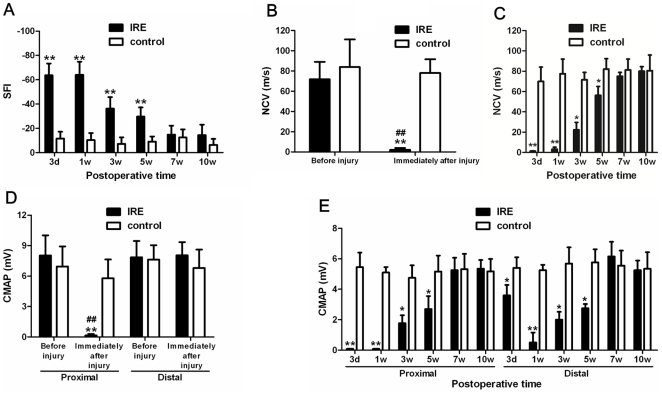
Functional recovery after sciatic nerve injury. (a) Measurements made from walking track prints were then submitted to SFI. (b) NCV evaluation before and immediately after sciatic nerve injury. (c) NCV evaluation at 3 days, 1 week, 3, 5, 7, and 10 weeks after sciatic nerve injury. (d) CMAP evaluation before and immediately after sciatic nerve injury. (e) CMAP evaluation at 3 days, 1 week, 3, 5, 7, and 10 weeks after sciatic nerve injury. *P<0.05, **P<0.01 versus control group.

3 days after injury, the sciatic functional index (SFI) value was significantly decreased in IRE compared with the control (*P*<0.01). 1 week after injury, the SFI value in IRE group was similar to that at 3 days after injury, and it was notably decreased compared with the control (*P*<0.01). Following several weeks after injury, the SFI values in IRE at 3 and 5 weeks after injury were increased, but these SFI values were still markedly decreased compared with the control (*P*<0.01). 7 and 10 weeks after injury, the SFI values were increased than before and there were no significant differences between IRE and the control (Figure 1A).

### Electrophysiological evaluation

The results showed that after injury, the nerve conduction velocity (NCV) was robustly reduced than that both before injury (*P*<0.01) and controls (*P*<0.01) (Figure 1B). 3 days and 1 week after injury, there were no restoration and the NCV were still significantly decreased compared with the control (*P*<0.01). Furthermore, the NCV restored following several weeks after injury. 3 and 5 weeks after injury, the NCV were elevated than that before, but still observably decreased compared with the control (*P*<0.05). 7 and 10 weeks after injury, the NCV were restoration, because there were no differences between IRE and the control (Figure 1C).

The proximal compound muscle action potential (CMAP) was also significantly decreased compared with both that after injury (*P*<0.01) and the control (*P*<0.01) (Figure 1D). However the distal CMAP was different from the proximal one. There were no differences not only between before and immediately after injury but also differences between IRE and the control immediately after injury (Figure 1D). 3 days after injury, the proximal CMAP was dramatically decreased compared with the control (*P*<0.01), but there was no significant difference between IRE and the control in the distal CMAP. 1 week after injury, the proximal CMAP was still significantly decreased compared with the control (*P*<0.01), while the distal CMAP was specifically lessened compared with the control (*P*<0.01). 3 and 5 weeks after injury, we find both the proximal and distal CMAP were augmented than that before, but still less than the control (*P*<0.05). 7 and 10 weeks after injury, both the proximal and dismal CMAP were marked restoration, and there were no differences between IRE and the control (Figure 1E).

### Histologic and Morphometric Analysis

**Figure 2:**
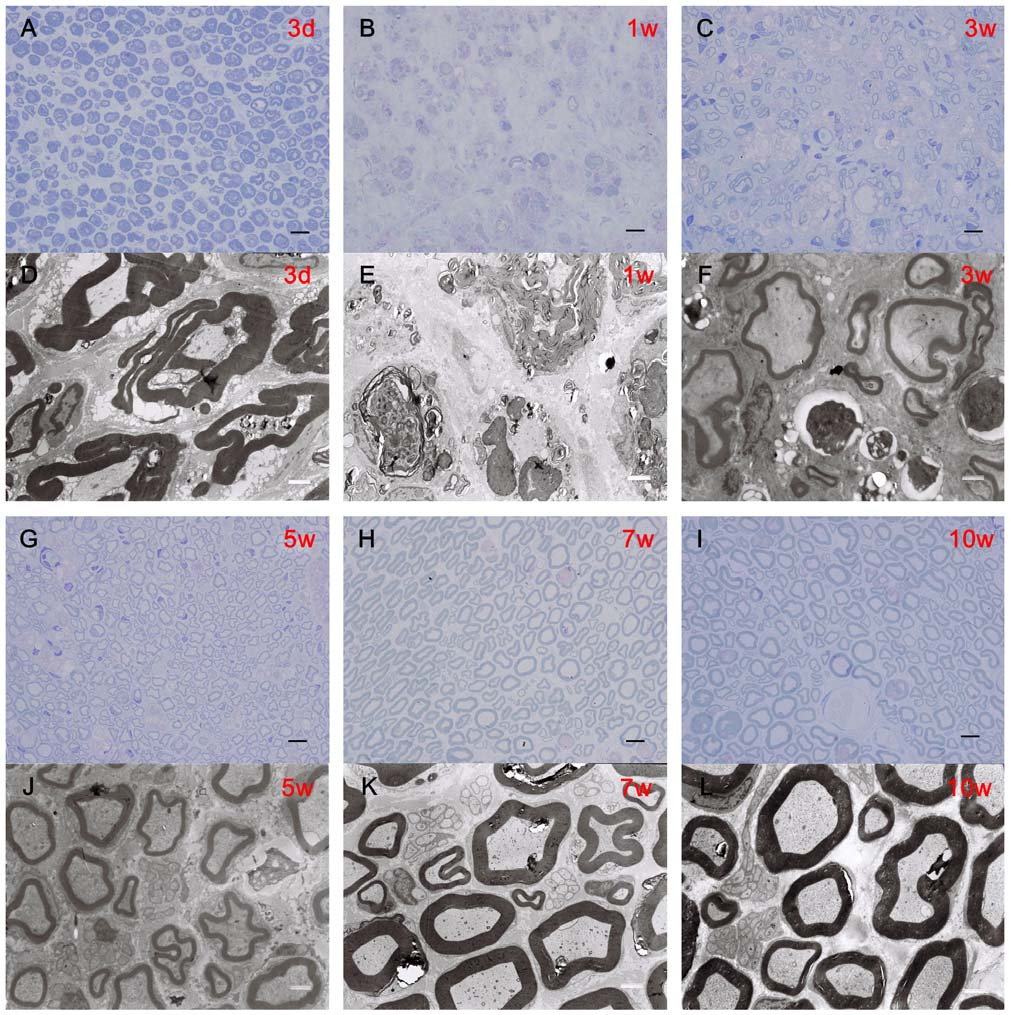
Remyelination of sciatic nerves. (a–c, g–i) Toluidine blue staining. Light micrographs of transverse semi-thin sections at the injury sites of IRE at 3 days, 1 week, 3, 5, 7 and 10 weeks after injury. (d–f, j–l) Transmission electron micrographs(TEMs). Ultra-thin sections at the injury sites of IRE at 3 days, 1 week, 3, 5, 7 and 10 weeks after injury were observed under TEM. **Scale bars**: A–C, G–I, 10 µm. D–F, J–L, 1 µm.

Transverse semi-thin sections at the injury sites of sciatic nerves were analyzed by Toluidine blue staining. We found that 3 days after injury, some of myelin sheath structures disintegrated and 1 week after injury, myelin sheath structures barely existed. Many blue fragments were observed at this time point. 3 and 5 weeks after injury, some myelin sheath structures have been restored, meanwhile some nerve fibers of regeneration appeared. 7 and 10 weeks after injury, many nerve fibers regenerated, and most of the myelin sheath structures have restored perfectly (Figure 2A, B, C, G, H, I).

Ultra-thin sections were observed under the transmission electron microscope. We found increasing numbers of vacuoles, some inflammation cells, and many folded myelin sheaths. 1 week after injury, many disintegrated myelin sheath were observed. Axons have disappeared but basal membrane was still integrated. 3 and 5 weeks after injury, there were still some disintegrated myelin sheath observed, but more and more nerve fibers of regeneration were surrounded by Schwann cells. 7 and 10 weeks after injury, most injury regions have restored. Myelin sheath structures were similar to the control and normal structures. Only seldom folded myelin sheaths were observed at this time point (Figure 2D, E, F, J, K, L).

**Figure 3:**
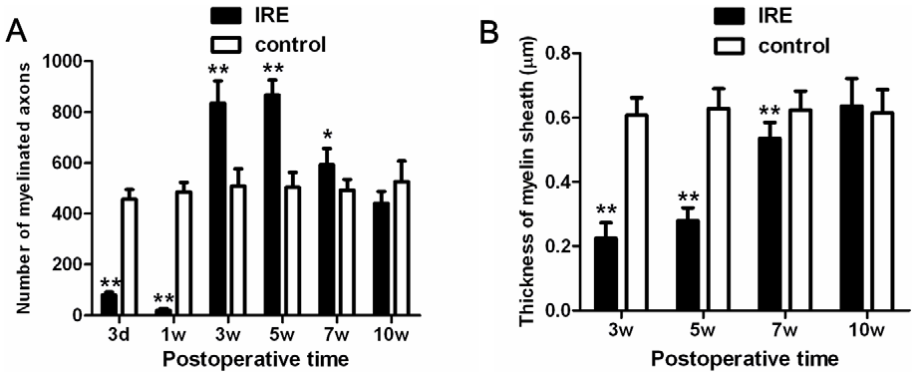
(a) The statistical analysis of the number of myelinated axons. (b) The statistical analysis of thickness of myelin sheath.

The statistical analysis revealed that at 3 days and 1 week after injury, the number of myelinated axons was notably decreased compared with the control (*P*<0.01). However, the number of myelinated axons was significantly elevated when compared with the control 3 and 5 weeks after injury (*P*<0.01). 7 weeks after injury, the number of myelinated axons decreased gradually, but still remarkably increased compared with the control (*P*<0.01). Finally, 10 weeks after injury, there was no significant difference between IRE and the control (Figure 3A). We also evaluated the thickness of myelin sheath. 3 days and 1 week after injury, the thickness of myelin sheath could not be measured because many myelin sheath fragments were observed and myelin sheath structures disappeared already. The statistical analysis revealed that 3 and 5 weeks after injury, myelin sheath of IRE was markedly thinner than that of control (P<0.01). 7 weeks after injury, the thickness increased than that before, but it still was significantly decreased compared with the control (P<0.01). The thickness of myelin sheath of IRE recovered and was similar to that of control at 10 weeks after injury (Figure 3B).

### Histological analysis of target muscle

**Figure 4:**
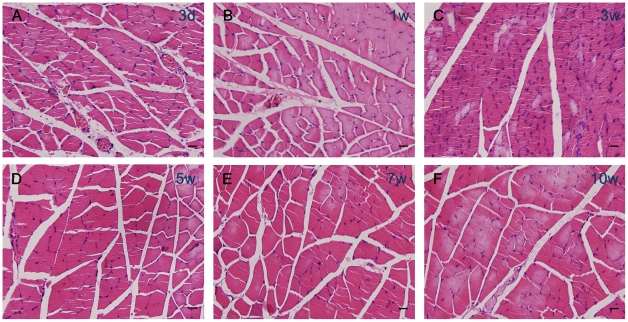
Histological analysis of target muscle. (a–f) Hematoxylin and eosin staining. Light micrographs of transverse sectioned gastrocnemius muscle on the IRE side at 3 days, 1 week, 3, 5, 7 and 10 weeks after injury. **Scale bars**: 10 µm.

**Figure 5:**
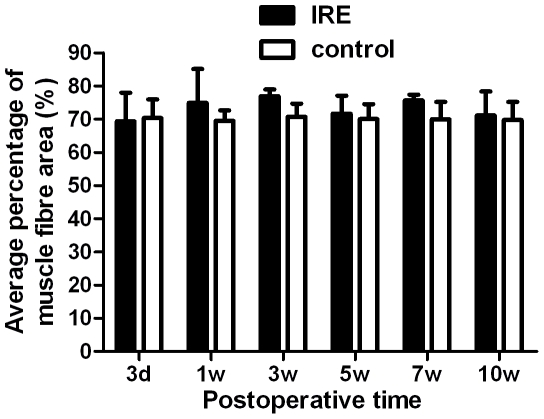
The statistical analysis of the average percentage muscle fiber area. There were no significant differences between IRE and the control.

The histological appearance of gastrocnemius muscles was assessed at 3 days, 1 week, 3, 5, 7 and 10 weeks after injury. There were no significant differences between IRE and the control at 3 days, 1 week, 3, 5, 7 and 10 weeks after injury in the average percentage of muscle fiber area (Figure 4. Figure5).

## Discussion

The typical parameters of irreversible electroporation are the sequence of 100 microsecond pulses. The cells that are exposed to an electrical field above 600 V/cm are ablated [8]. We had applied IRE (3800 V/cm) directly to the sciatic nerve of rats. The electrical field was very high on the scale of typical IRE pulses, so the area tissue should be totally ablated. Considering the nerve function of the control group is preserved, the injuries to nerve are presumed to be related to the IRE, not a mechanical crushing of the nerve. After the peripheral nerve sustains direct IRE injury, the pathophysiologic changes are typical Wallerian degeneration and regeneration of axons. Our results are supported by the works of Onik, G. Rubinsky, B. et al [14], [16]. These results show that at 2 weeks nerves appeared to be intact and unaffected [14]. Intact nerve bundles with viable ganglion cells were observed after 3 weeks [16]. Morphologic modifications of the nerve were only examined for the first 3 weeks after IRE ablation. The long-term morphologic modifications and function evaluation had not been assessed. The aim of this study was to evaluate the long term effect of IRE on nerves.

The current gold standard for bridging nerve gaps is nerve autografting. Reviewing the published results of 10 mm autograft, the SFI on Day 60 were respectively −79.9 and -76.74 [17], [18]. None of the repair methods including nerve autografting were able to restore SFI parameters to control levels [18]. Our results show the SFI of the IRE treatment group returns to control levels after 7 weeks. Obviously, nerve regeneration after IRE ablation was able to obtain better results. Electrophysiological, histological, and functional studies show the nerve treated with IRE ablation can attain full recovery. The ideal thing is to spare the nerve in radical surgery of resecting the malignant tumors involved critical nerve. Unfortunately, this has been rarely possible in the past. Emergence of IRE ablation may make it possible. The patient may suffer a temporary nerve function loss and be able to regain total nerve regeneration.

The most widely used tumor ablation method is thermal ablation. Thermal ablation creates coagulative necrosis and leaves necrotic tissue, which are toxic to the organs [10]. Those nerves in thermal ablative zones cannot regenerate, whereas our results show that the nerve after IRE can achieve complete recovery. Several unique characteristics of IRE distinguish itself from other current tumor ablative techniques. These characteristics might be the causes of perfect nerve regeneration. (1) Because the structures which mainly formed by proteins are not damaged by IRE ablation [10], IRE treatment does not affect the basal lamina or endoneurial integrity but the axon is damaged. The injuries are similar to second-degree injury (Sunderland) or axonotmesis. The endoneurium and perineurium remain intact and therefore facilitate nerve regeneration. Regenerating axons follow their normal course and arrive at their original target, so the prospect of recovery is excellent in such injuries [19]. Our results also show complete recovery. The severe injury to axon could induce neural cell death. Neuron survival after axonotmesis is a prerequisite for regeneration [20]. According to the perfect regeneration of nerve after axon IRE injury, we can draw a conclusion that neural cell survival is maintained. Muscle innervated by the sciatic nerve were observed prominently contracting in all animals treated with IRE. After 7 weeks, those muscles recovered normal functions. All animals in our experiments survived. Our results support that IRE is a safe ablation method for clinical application [21]. (2) The IRE is a non-thermal ablative technique, the electric field created by IRE is devoid of any joule heating and therefore, non-thermal necrosis is occurred [22]. Shafiee, Garcia et al had provided an example that determines the upper limit of irreversible electroporation (onset of thermal damage) through a theoretical calculation [23]. We chose an electric field of 3800 V/cm which is below the upper bound of thermal damage. We didn’t observe the thermal burn, and another research used the same electrical field support our results [9]. IRE causing complete tissue death by means of apoptosis [24], [25] or “apoptosis-mimetic” necrosis [26]. This feature has many beneficial effects. It allows extremely rapid regeneration of ablated tissue [8]. Schwann cells are a key factor in nerve regeneration. Not only did they have a role in phagocytosis of myelin, but they also have a role in producing neurotrophic factors. Though the Schwann cells within the ablative zone were killed, Schwann cells were observed and the normal myelin sheath structures were restored in ablative segment. Those cells might be recruited to the injury site. (3) We applied IRE directly to nerve. Minimal endothelial damage in small vessels of the nerve was observed in the early phase. After 3 weeks, the damaged vessels become normal form with an intact endothelium. The preservation of the microvasculature increases the potential of nerve regeneration in the area of ablation.

We chose an electric field of 3800 V/cm and 10 pluses as our ablative protocol. This was based on the study of the effects of IRE on a large blood vessel [9]. The subsequent study shows that ten pulses of 3500 V/cm achieved a similar effect to 90 pulses of 1750 V/cm [27]. It seems that our protocol and a typical IRE protocol can cause the same effects. However, further studies are needed in order to better understand the long term effect of different IRE protocols on nerves. We choose the most commonly used rat sciatic nerve model. Though the rats are small, their nerves regenerate well [28]. We have to choose large animals in the process from preclinical study to clinical application. Larger animal nerve regeneration processes have been shown to be more similar to humans, and large animals could be used to assess longer distance nerve regeneration [29], [30]. These large animal studies are necessary to evaluate the possibility of applying IRE to clinical nerve-sparing tumor ablation in humans.

This is a pilot study on the effects of IRE on nerves. Considering that we have treated a substantial length of a rat sciatic nerve with direct, high electrical field IRE pulses, and the nerve was able to demonstrate a full functional recovery, this finding is indicative of the preserving of nerve involving malignant tumors with respect to the application of IRE pulses to ablation tumors completely. In summary, IRE could be a promising treatment tool for any tumor involving nerves.

## Materials and Methods

### Animals and grouping

All the experimental procedures involving animals were conducted under a protocol reviewed and approved by the Ethics Committee of Tangdu Hospital, Fourth Military Medical University (approval ID:2010028). Young adult female Sprague-Dawley rats (n = 60, provided by the Laboratory Animal Center of the FMMU, Xi’an, China) weighing 200 g to 220 g were randomized into 2 groups with 30 animals each: group I(IRE) and group II (Control).

### Surgical procedures and irreversible electroporation

**Figure 6:**
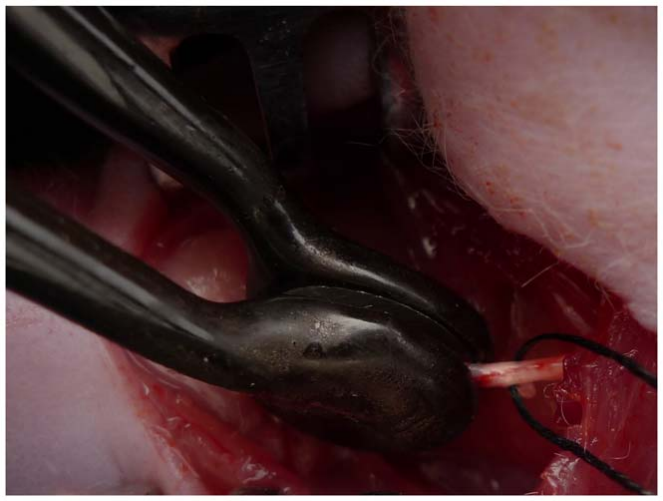
The IRE device clamping the exposed right sciatic nerve.

The animals were anaesthetized by an intraperitoneal injection of sodium pentobarbital solution (10 mg/mL, 40 mg/kg body weight) and the hair on the right femur was removed. Under aseptic conditions the right sciatic nerve was exposed by making a skin incision and splitting the underlying muscles in the right lateral thigh. At least 15-mm long segment of sciatic nerve was ready for electroporation. A specially designed hand-held clamp, containing two parallel metal electrodes (BTX, USA), was applied directly on the sciatic nerve(Figure 6). The distance between the electrodes measured with a caliper to be approximately 1.0 mm. A sequence of 10 direct current square pulses of 380 V (generating an approximate electrical field of 3800 V/cm), each 100 µs long, was applied between the electrodes using an electroporation pulse generator ECM2001 (BTX, Cambridge MA) [9]. In each animal of group I(IRE) the procedure was applied to produce a treated length of about 10 mm. In each animal of group II(Control) the electrodes were only applied directly on the sciatic nerve for the same time. Keep the distance of the electrodes approximately 1.0 mm. There was no crush injury by the electrodes, for sciatic nerves were about 1.2 mm. Injury level was marked by 9/0 non-absorbable suture. At the end of the operation the muscle and skin were sutured. All surgeries were performed by the same surgeon. After operation, the animals were kept separately. Tests were performed on immediately after and 3 days, 1 week, 3, 5, 7 and 10 weeks following surgery.

### Behavioral analysis

We assessed the functional nerve recovery following sciatic nerve injury using rat walking track analysis by recording its footprints. Walking track analysis was performed on all the animals before surgeries. At 3 days, 1 week, 3, 5, 7, 10 weeks after nerve injury the animals were submitted to walking track analysis and measurement of the sciatic functional index (SFI) using a method as described previously [31], [32] with minor modifications. The rats were allowed conditioning trials in an 8 cm×50 cm walking track with a piece of white paper at the bottom of the track with two to three times repeat. The left hind feet were dipped in blue ink and the right hind feet were dipped in red ink, leaving prints on the white paper. Any changes in their paw prints that resulted from nerve injury and denervation were recorded. The clear and integral footprints were surveyed until 6 to 8 measurable footprints collected. We could calculate the SFI based on the following formula [31]:
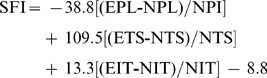



Where EPL indicated the operated experimental paw length; NPL, normal paw length; ETS, operated experimental toe spread, i.e., the distance between the first and fifth toes; NTS, the normal toe spread; EIT, the operated experimental intermediary toe spread, i.e., the distance between the second and fourth toes; and NIT, normal intermediary toe spread. The SFI was scaled such that -100 represented the sciatic nerve was injured completely or complete loss nerve function and 0 represented normal function or completely recovery. SFI of 0±11 was also set as normal nerve function [33].

### Electrophysiological testing

Electrophysiological studies were performed before, immediately after and 3 days, 1 week, 3, 5, 7, 10 weeks after surgery, prior to tissue harvesting. In this experiment we evaluated two parameters, nerve condition velocity (NCV) and compound muscle action potential (CMAP). After anesthesia was induced, the sciatic nerve was exposed. A recording electrode was placed in the gastrocnemius muscle and the compound muscle action potential (CMAP) was recorded. The CMAP and NCV values were calculated [34]. Bipolar stimulating and record electrodes were used to induce and record electrical activity. We recorded NCV and CMAP in responding to the stimuli using neuromax 1004 (XLTEK, Canada).

### Morphometric analysis

Morphological assessments were performed at 3 days, 1 week, 3, 5, 7, and 10 weeks after sciatic nerve injury. After the sciatic nerves (about 10 mm) at the injury sites were harvested and fixed in 3% glutaraldehyde, the samples were postfixed in 1% osmium tetroxide in 0.1 M sodium cacodylate buffer (PH 7.3) for 1 hour at room temperature, dehydrated in ethanol, and embedded in resin. Transverse semi-thin (thickness 1.0 µm) and ultra-thin (thickness 50.0 nm) sections were prepared. The semi-thin sections were stained with a 1% toluidine blue and examined under a light microscope (BX51, Olympus, Tokyo, Japan). The number of myelinated axons was counted from at least 6 randomly selected fields under the magnification of ×1000 and analyzed with Image-Pro Plus software (Media Cybernetics). Ultra-thin sections were stained with uranyl acetate and lead citrate and were examined under a transmission electron microscope (H-600, HITACHI, Tokyo, Japan).

### Histological analysis of target muscle

To measure the target muscle reinnervation, after the animals had been killed, the gastrocnemius muscles from the operated limbs of rats were removed. Several pieces of the excised gastrocnemius muscle were cut from its mid-belly and post-fixed in 4% paraformadehyde. The fixed muscle specimens were embedded in paraffin and cut into 5 µm-thick sections, to which hematoxylin and eosin (HE) staining was applied before photographs were taken. For each HE stained section from every specimen, photographs were taken from 3 random fields and analyzed with Image-Pro Plus software (Media Cybernetics) to measure the transverse section area of the muscle fibers. The percentage of muscle fiber area was calculated as muscle fiber area/total area.

### Statistical analysis

All data were expressed as means ± standard deviation. Statistical differences between groups were analyzed by two-tailed student’s *t*-test with the SPSS13.0 software package (SPSS Inc, Chicago,IL). Differences were considered statistically significant as * *P*<0.05, ** *P*<0.01.
